# Editorial for the Special Issue on Droplet-Based Microfluidics: Design, Fabrication, and Applications

**DOI:** 10.3390/mi14030693

**Published:** 2023-03-21

**Authors:** Pingan Zhu

**Affiliations:** Department of Mechanical Engineering, City University of Hong Kong, Hong Kong 999077, China; pingazhu@cityu.edu.hk

Microfluidics is a rapidly growing field of research that involves the manipulation and analysis of fluids in small-scale channels, usually with dimensions ranging from sub-micrometer to sub-millimeter. This technology has widespread applications in fields such as chemistry, biology, medicine, physics, engineering, and the environment. One particularly appealing subcategory of microfluidics is droplet-based microfluidics, which permits the generation, manipulation, and analysis of tiny droplets and bubbles with exceptional precision and accuracy. Advancing this field necessitates innovative techniques for droplet/bubble generation and manipulation, as well as the development of novel materials and technologies for microfluidic devices. The aim of this Special Issue is to showcase the latest developments in droplet-based microfluidics, with a focus on the fundamentals of fluid mechanics, fabrication of microfluidic devices, and the generation, manipulation, and applications of droplets. The fundamentals of fluid mechanics continue to be important in the design and optimization of microfluidic devices, while advances in fabrication techniques have enabled the creation of increasingly complex and precise devices. Droplet generation and manipulation are also critical for many microfluidic applications, ranging from materials synthesis to chemical and biological analysis.

## 1. Fundamentals of Fluid Mechanics

Fluid mechanics is the cornerstone of droplet-based microfluidics as it governs the behavior of fluids at the microscale. A comprehensive comprehension of the principles of fluid mechanics is vital to devise and create effective droplet-based microfluidic systems.

Frolov et al. [1] explore the interaction of shock waves with bubbly water for generating a propulsive force. The study aims to investigate two potential directions for improving underwater propulsion: (1) replacing chemically inert gas bubbles with chemically reactive ones and (2) increasing the pulsed detonation frequency from tens of hertz to kilohertz. This study could offer valuable insights into the behavior of bubbles in microfluidic devices that utilize shock waves, thereby paving the way for designing microfluidic devices that leverage shock waves for various applications, including droplet generation and manipulation.

In addition to bubble dynamics, Frolov et al. [2] investigate the self-ignition of triethylaluminum (TEA) and triethylborane (TEB) microdroplets in air. The authors propose a novel mechanism of the heterogeneous interaction of gaseous oxygen with liquid TEA/TEB microdroplets to calculate the self-ignition of a spatially homogeneous mixture of fuel microdroplets in ambient air under normal pressure and temperature conditions. The findings provide insights into the combustion behavior of fuel droplets, which can aid in designing microfluidic devices that involve reactions with highly reactive droplets for micro-combustion.

Concerning solid–fluid interactions, Sheidaei et al. [3] present a numerical study that predicts the dispersion rate of nanoparticles in a gas–liquid dual-microchannel separated by a porous membrane. The dispersion rate of airborne nanoparticles can be regulated by adjusting the fluid flow velocity, membrane porosity, and particle diameter. This study offers fundamental insights into the mechanisms of nanoparticle dispersion, which can be utilized to optimize the design of lung-on-a-chip microfluidic devices for nanoparticle separation and filtration with implications for health monitoring.

## 2. Fabrication of Microfluidic Devices

The fabrication of microfluidic devices requires an understanding of material properties and manufacturing techniques that can be used to create structures with desired characteristics. Atmakuri et al. [4] explored the effect of filler materials on the wettability and mechanical properties of basalt/E-glass woven fabric-reinforced composites, which could be used to create microfluidic devices with high mechanical strength and tunable wettability. They found that the incorporation of graphite improves both the hydrophobicity and mechanical properties of the composites.

In the field of 4D printing, Long et al. [5] developed variable stiffness conductive composites that can change their stiffness and conductivity properties in response to external stimuli by alternately printing liquid metals and silicone elastomers. This technology has potential applications in microfluidics, where materials with programmable properties can be used to fabricate droplet-based microfluidic devices with on-demand manipulation of droplets and fluid flows.

Thermal imprinting is a common technique for creating microfluidic structures with high aspect ratios, but the accuracy of microstructures can be compromised due to the thermal behavior of the materials during imprinting. Ciganas et al. [6] developed a finite element model for accurately predicting the thermal imprint of high-aspect-ratio microfluidic structures fabricated from different polymers, providing insights into the behavior of these materials during imprinting. The models can provide guidelines for optimizing imprinting conditions and parameters, which is conducive to the fabrication of microfluidic devices with improved accuracy of microstructures.

Finally, Cai et al. [7] develop a method for the fabrication of transparent and flexible digital microfluidic devices with improved optical properties and mechanical flexibility by laser ablation. The device can perform different droplet manipulation functions and provides an alternative to conventional digital microfluidic devices that are based on glass or silicon wafers.

## 3. Droplet Generation

The fabrication of microfluidic devices for droplet generation has been a major focus of recent research in the field. Anyaduba et al. [8] demonstrate a novel approach to generating picoliter-sized droplets with high precision and efficiency using microfluidic devices fabricated via 3D printed molds. By utilizing complex geometries made possible by 3D printing, the authors provide a promising avenue for high-throughput biological or chemical assays.

The control of droplet generation in microfluidic devices heavily relies on the surface wettability of microfluidic channels. In this regard, Warr et al. [9] investigate the surface modification of 3D printed microfluidic devices to achieve controlled wetting in two-phase flow. By utilizing hydrophobic monomers, the authors have developed a technique to render the surfaces of microfluidic devices more hydrophobic, providing a means to regulate droplet generation in microfluidic devices. This advancement in surface modification technology is expected to have significant implications in the field of chemical and biological assays.

Dai et al. [10] delved into the dynamic behavior of double emulsion formation in a tri-axial capillary device. The study provides insights into the mechanisms of double emulsion generation in microfluidic devices by developing a semi-analytical model for predicting the droplet size distribution with the wall effect and various flow conditions. By utilizing a one-step process and controlling the size of the droplets, the authors provided a promising avenue for monodisperse double emulsion generation in microfluidic devices.

Trossbach et al. [11] presented a portable, negative-pressure actuated, dynamically tunable microfluidic droplet generator. The authors demonstrate the capabilities of the device by using it to produce monodisperse droplets with varied volumes, dynamically tune the droplet composition, and create droplet-templated cell spheroids from primary cells. This device’s portability and easy-to-use nature make it a valuable tool for a range of applications, especially for non-specialists.

## 4. Droplet Manipulation

Microfluidic droplet manipulation is a rapidly growing field that has potential applications in various scientific and technological domains. In this regard, researchers have focused on investigating the fundamental mechanisms behind droplet manipulation and exploring new techniques for precise control over droplet generation, sliding, and transfer.

Chen et al. [12] present a study on the asymmetric jetting phenomenon that occurs during the impact of liquid drops on superhydrophobic concave surfaces. The study reveals that the deformation of the liquid-liquid interface during the droplet impact causes asymmetric jetting, with the droplet’s impact position and surface curvature playing crucial roles. These findings provide insights into the underlying mechanisms of droplet manipulation and have significant implications for designing open-space microfluidic devices with controlled droplet manipulation and generation.

Yonemoto et al. [13] investigate the sliding behavior of droplets on an inclined solid substrate. The study highlights the dependence of the onset of droplet sliding on factors such as droplet size, substrate inclination angle, and contact angle. The results contribute to the fundamental understanding of droplet manipulation mechanisms and provide a basis for designing open-space microfluidic devices in applications such as droplet-based assays and droplet-based microreactors.

In addition to droplet impact and sliding, precise control over the amount and location of liquid deposition is another critical aspect of droplet manipulation. Liu et al. [14] present a novel technique that utilizes a micropipette to transfer ultra-small volumes of liquid adhesive onto a substrate in the femtoliter to picoliter range. The method enables precise control over the amount and location of adhesive, opening up new avenues for designing and developing microfluidic devices with controlled material distribution.

## 5. Applications of Droplet-Based Microfluidics

Droplet-based microfluidics has found numerous applications in various fields, including medical diagnostics, materials science, and wearable electronics. In this regard, Tiemeijer et al. [15] present a single-cell droplet microfluidics platform for analyzing the functional heterogeneity of cytotoxic T-cells (CTLs). The platform utilizes soluble stimuli and artificial antigen-presenting cells (APCs) to activate CTLs and identify functional heterogeneity based on various parameters. The tool proposed provides a means for high-throughput and single-cell analysis of CTLs, paving the way for the selection of potent CTLs for cell-based therapeutic strategies.

In continuous crystallization processes, the suitability of material systems is critical to achieving high-quality crystals. Kufner et al. [16] propose a strategy for fast decision making on the suitability of material systems for continuous crystallization using a microfluidic slug flow crystallizer. The approach involves pre-selection of the solvent/solvent mixture, verifying slug flow stability, and modeling temperature-dependent solubility in the material system. The strategy represents a general approach for optimizing the design and operation of continuous crystallization processes.

Choe et al. [17] present a novel wearable strain sensor that utilizes droplet-based technology. The sensor is made of ultrasoft and ultrastretchable silicone elastomers filled with conductive liquid-metal droplets, exhibiting anisotropic conductivity, and maintaining metallic conductivity when strained. The sensors can be integrated into clothing and conform to the body, making them suitable for use in healthcare and sports applications, including the development of wearable electronics and soft robotics.

In summary, the articles presented in this Special Issue showcase the extensive range of research being conducted in the field of droplet-based microfluidics and emphasize the significance of droplet-based microfluidics as a versatile and robust tool for scientific research and technological advancement. From fundamental studies of fluid mechanics to the design of innovative devices and their applications in various fields, droplet-based microfluidics provides a plethora of exciting research opportunities. Further exploration of this field will undoubtedly uncover new discoveries and applications in the near future.

Lastly, I extend my sincere gratitude to all the authors for their valuable contributions to this Special Issue, and to the reviewers for their dedicated efforts and time spent enhancing the quality of the papers.
